# 894. One-year mortality and morbidities of severe fever with thrombocytopenia syndrome compared with other diseases: a nationwide cohort study in South Korea

**DOI:** 10.1093/ofid/ofad500.939

**Published:** 2023-11-27

**Authors:** Namwoo Heo, Seok-Jae Heo, Yoon Soo Park, Seonju Yi, Yong Chan Kim

**Affiliations:** Yongin Severance Hospital, Yonsei University College of Medicine, Seongnam-si, Kyonggi-do, Republic of Korea; Yonsei University College of Medicine, Seoul, Seoul-t'ukpyolsi, Republic of Korea; Department of Internal Medicine, Division of Infectious disease, Yongin Severance Hospital, Yonsei University College of Medicine, Yongin, Kyonggi-do, Republic of Korea; Korea Disease Control and Prevention Agency, Seoul, Seoul-t'ukpyolsi, Republic of Korea; Department of Internal Medicine, Division of Infectious disease, Yongin Severance Hospital, Yonsei University College of Medicine, Yongin, Kyonggi-do, Republic of Korea

## Abstract

**Background:**

Severe fever with thrombocytopenia syndrome (SFTS) is a tick-borne disease with high mortality. However, most studies have focused on the short-term mortality of patients with SFTS, and little is known about long-term outcomes in patients with SFTS.

**Methods:**

This retrospective cohort study was conducted using the National Health Insurance Service dataset on hospitalized patients with SFTS aged ≥20 years between 2016 and 2021 (n=1,217). Each SFTS case was matched with three controls hospitalized for non-SFTS-related diseases using propensity score matching. All-cause mortality was evaluated in patients with SFTS during 1-year follow-up and compared with controls. Post-discharge events were investigated to determine the effects of SFTS on post-acute sequelae.

**Results:**

Finally, 1,105 patients with SFTS and 3,315 controls were included. Patients with SFTS had a higher risk of death during the 1-year follow-up than controls (hazard ratio [HR], 2.26; 95% confidence interval [CI], 1.82–2.81). Mortality in the first 30 days was significantly higher in the SFTS group (HR, 3.99; 95% CI, 3.07–5.19) than in the control group. An increased risk of death after 31–365 days was observed in the control group; however, statistical significance was identified only in patients in their 80s (HR, 0.18; 95% CI 0.06–0.57). For the post-discharge events, the SFTS group showed a higher risk of readmission (HR, 1.17; 95% CI, 1.04–1.32) and emergency room visit (HR, 2.32; 95% CI, 1.96–2.76) than the control group.

**Figure:**
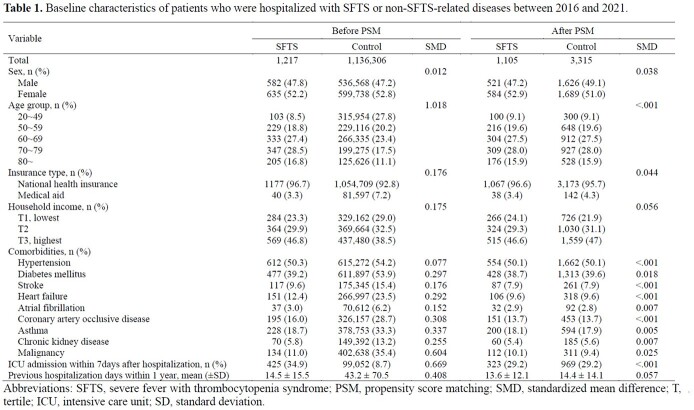

**Figure:**
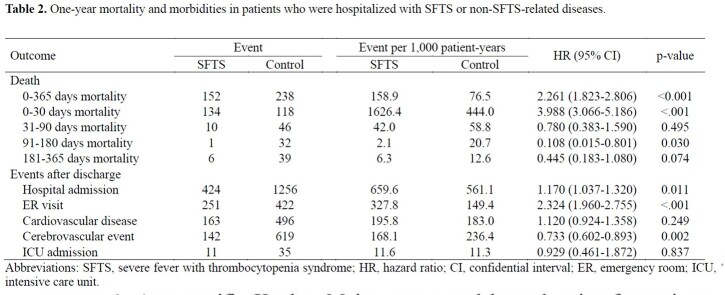

**Figure:**
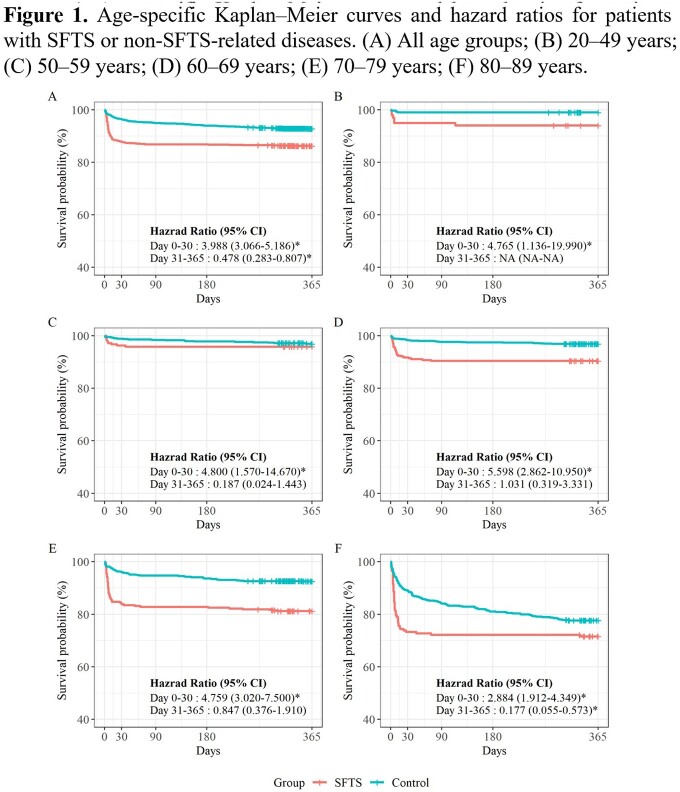

**Conclusion:**

SFTS is associated with a higher risk of short-term mortality and post-acute sequelae in hospitalized patients during 1-year follow-up than non-SFTS-related diseases. Our results provide evidence for the management of patients with SFTS.

**Figure:**
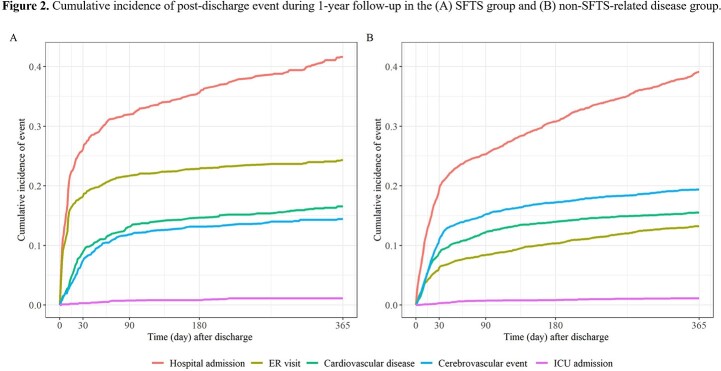

**Disclosures:**

**All Authors**: No reported disclosures

